# Transforming critical care: the digital revolution's impact on intensive care units

**DOI:** 10.3389/fdgth.2025.1664382

**Published:** 2025-11-21

**Authors:** Corina Vernic, Tudor Paul Tamas, Ion Petre, Sorin Ursoniu

**Affiliations:** 1 Doctoral School, Victor Babes University of Medicine and Pharmacy Timisoara, Timisoara, Romania; 2 Department of Functional Sciences III, Discipline of Medical Informatics and Biostatistics, Victor Babes University of Medicine and Pharmacy, Timisoara, Romania; 3 Research Center CCATITM, Faculty of Medicine, Victor Babes University of Medicine and Pharmacy, Timisoara, Romania; 4 Department of Functional Sciences III, Discipline of Physiology, Victor Babes University of Medicine and Pharmacy, Timisoara, Romania; 5 Department of Functional Sciences III, Discipline of Public Health and History of Medicine, Victor Babes University of Medicine and Pharmacy, Timisoara, Romania; 6 Centre for Translational Research and Systems Medicine, Faculty of Medicine, Victor Babes University of Medicine and Pharmacy, Timisoara, Romania

**Keywords:** ICU care metrics, machine learning, digitalization, quality and safety, indicators and metrics

## Abstract

Intensive care units (ICUs) represent a critical pillar of modern healthcare, combining advanced technologies and specialized care to support organ functions in critically ill patients. The recent COVID pandemic served not only as a stress test but also as a potential catalyst for further ICU digitalization advancements. Recently evolved tools and processes suggest a transformative potential for digitalization in enhancing ICU performance, optimizing resource utilization, and improving patient outcomes. Digital tools—particularly machine learning (ML) and artificial intelligence (AI)—could significantly support ICU care by facilitating real-time monitoring, predictive analytics, and semiautomated decision-making. ML models have shown promise in outperforming traditional scoring systems when predicting patient outcomes such as mortality, ICU length of stay, and readmission risks. The digitalization of nursing documentation and resource allocation processes appears to improve efficiency, reduce errors, and potentially optimize staff time for direct patient care. Innovations in infection control are increasingly leveraging AI to predict conditions like ventilator-associated pneumonia and sepsis, enabling earlier interventions and potentially enhancing antimicrobial stewardship. Closed-loop ventilation systems illustrate a shift toward intelligent, data-responsive care platforms that may improve patient safety and therapeutic precision by embedding adaptive decision-making into medical devices. The pandemic underscored the growing relevance of ICU digitalization, accelerating the development of tools such as remote monitoring, tele-ICU models, and wearable devices. These advancements have helped address unprecedented patient volumes and further illustrated the potential of AI-enabled tools to streamline ICU workflows and augment patient care. This momentum reflects a broader paradigm shift in critical care toward more proactive, algorithm-assisted medicine—where AI is positioned to complement clinical judgment in managing the complexity of ICU environments. In addition, personalized digital recovery pathways are being explored to support post-ICU rehabilitation, although significant challenges remain in addressing patients’ physical and psychological recovery needs. Altogether, these recent evolutions underscore the potentially transforming role of digitalization in enhancing ICU care quality and safety parameters, improving resource utilization, supporting better patient outcomes, and helping meet the evolving expectations of patients and their families.

## 1 Introduction

The intensive care unit (ICU) functions as a highly specialized, patient-centered component within the broader healthcare framework, designed to manage critical physiological instability with precision and responsiveness. At its core, it integrates complex technological infrastructures with the coordinated efforts of a multidisciplinary workforce trained to navigate acute, rapidly evolving clinical scenarios. These teams often act as adaptive agents, dynamically interpreting continuous physiological data to recalibrate therapeutic interventions in real time. The ICU can thus be viewed as a feedback-rich environment where the primary objective remains the stabilization and recovery of organ systems in crisis, frequently due to trauma, sepsis, or postoperative complications. This reflects the ICU role as a potentially vital life-sustaining node within the healthcare network.

The global COVID-19 crisis served as a stress test for ICU systems, revealing both their indispensable functionality and underlying vulnerabilities when faced with extreme demand. The surge in critically ill patients, especially those requiring invasive respiratory support, transformed ICUs into high-pressure operational hubs, where resource allocation, staffing scalability, and infrastructure flexibility were continuously strained. This situation underscored the ICU not merely as a clinical unit, but as a central element of public health strategy, emphasizing the value of robust surge capacity, inter-hospital coordination, and integrated emergency preparedness. In this context, the ICU could be interpreted as a microcosm of systemic healthcare resilience, reinforcing the importance of strategic investment in critical care infrastructure as a means of supporting population-level health (1).

The COVID-19 pandemic also acted as a catalyst for structural adaptation within the ICU ecosystem, accelerating the uptake of remote patient monitoring, tele-ICU platforms, and real-time data analytics into critical care operations. These technologies may serve as systemic extensions that augment the ICU's capacity to manage increasing patient volumes by decentralizing clinical oversight and enabling asynchronous collaboration across care teams. In doing so, they could improve operational efficiency and help mitigate the risk of staff fatigue, contributing to the maintenance of care quality during crisis conditions. The deployment of such digital infrastructures may not only allow for more agile system responses but could also reshape care delivery pathways by embedding resilience and scalability as key features of ICU systems better prepared for future demands. This experience highlighted the ICU's potential for both rapid adaptation and sustained innovation, reinforcing its strategic relevance in public health preparedness and broader systemic transformation (2, 3).

## 2 ICU care metrics

The digital revolution is transforming ICU care by enhancing efficiency, precision, and responsiveness across critical domains, from patient outcomes to safety. In patient outcomes, real-time monitoring and predictive analytics help detect health changes early, improving survival and recovery rates. Digital tools streamline clinical processes, ensuring swift interventions and protocol adherence through automated alerts and decision-support systems. Resource utilization benefits from digital scheduling, equipment tracking, and predictive maintenance, optimizing bed management and staff allocation. Patient and family satisfaction is elevated through communication platforms that keep families informed and engaged in care decisions. Finally, quality and safety indicators are strengthened with data-driven insights, reducing infections, adverse events, and unnecessary ventilator days, ultimately creating a safer, more efficient ICU environment (4).

The key performance parameters in ICU assessment focus first on patient-related outcomes, including mortality rate, length of stay (LoS), readmission rate, complication rate, and functional status after ICU. Mortality rates are assessed both within the ICU and hospital-wide, giving insight into the effectiveness of critical care within the healthcare provider unit. LoS measures the average duration patients spend in the ICU, with shorter stays generally reflecting efficient and effective care. Readmission rates within 48–72 h after discharge highlight cases where further critical care may be required, often signaling areas for improvement. Complication rates track the incidence of ICU-specific issues such as infections and ventilator-associated pneumonia, both of which directly impact recovery. Functional status after ICU assesses patients’ quality of recovery, focusing on their overall health and ability to return to daily activities (5, 6).

In medical care, adherence to established protocols is essential for maintaining high standards of care. Compliance with clinical guidelines for managing sepsis, ventilation, and infection control directly impacts patient outcomes and reduces the risk of complications. The timeliness of interventions is equally critical, ensuring life-saving treatments are administered swiftly and accurately. Medication and fluid management are monitored for accuracy and timeliness, as precise dosages and fluid balances are crucial in the ICU, where patient conditions can change rapidly (7, 8).

Resource utilization metrics evaluate how ICU resources are allocated, aiming for maximum efficiency without compromising care quality. Bed occupancy rates track the percentage of ICU beds in use, providing insight into demand and capacity. Staffing levels and skill mix refer to the ratio of trained ICU staff to patients, with a particular focus on nurse-to-patient ratios to ensure adequate support. Effective equipment and supply management is also vital, as it ensures that essential ICU equipment is available and well-maintained to handle critical situations (9, 10).

Patient and family satisfaction is another core performance area, encompassing family communication and end-of-life care. Frequent, high-quality updates and counseling sessions help families stay informed and support decision-making during a stressful period. End-of-life care quality is also crucial, providing families with compassionate support and guidance during critical or terminal phases, thus improving their overall ICU experience and easing emotional strain (11, 12).

**Figure 1:**
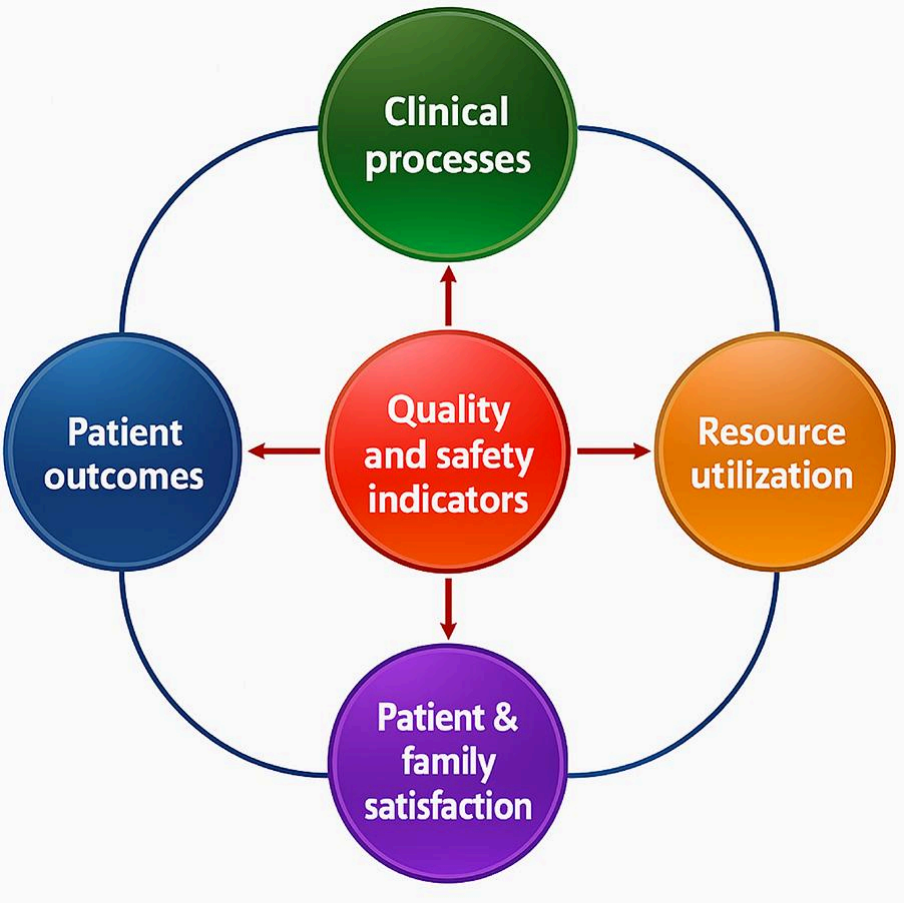
Overview of the key domains identified in ICU care.

Quality and safety indicators are key to ICU performance assessment (Figure 1), focusing on infection control, adverse events, and ventilator days. Infection control involves tracking healthcare-associated infections to prevent and manage them effectively. Adverse events, including medication errors, falls, or other preventable incidents, are closely monitored to improve safety protocols and reduce risk. Monitoring ventilator days helps prevent complications associated with prolonged ventilation, supporting better outcomes and reducing ICU stay duration (4, 13, 14).

This work aims to address the integration of digital technologies into ICU operations, particularly regarding the translation of machine learning (ML) and artificial intelligence (AI) tools into practical, system-level improvements in patient care, workflow, and resource management, using the ICU care metrics defined above. Although previous literature has explored isolated applications of ML and AI in critical care, such as mortality prediction or infection surveillance, this paper synthesizes these innovations into a framework that redefines the ICU as a cyber-physical system. It moves beyond proof-of-concept studies by consolidating real-world evidence on how digitalization affects key ICU care domains: patient outcomes, readmission prediction, documentation efficiency, infection control, ventilation optimization, and postdischarge recovery.

## 3 ICU digitalization impact on patient-related outcomes

Recent advances in ICU digitalization, particularly the use of ML and electronic health records (EHRs), have shown promising potential to improve patient outcomes by enhancing mortality prediction and optimizing care delivery. Complementary research demonstrates that digital interventions such as automated nursing documentation and computerized physician order entry (CPOE) may indirectly reduce mortality, by minimizing errors and increasing clinical efficiency.

### 3.1 Mortality and morbidity trends

A South Korean study evaluated the impact of ML models using electronic health records on predicting in-hospital mortality in ICU patients, comparing their performance with traditional scoring systems. Conventional models like APACHE III and SAPS III demonstrated moderate predictive accuracy, with area under the receiver operating characteristic curve (AUROC) values of in the range of 0.77–0.80. However, ML models, particularly XGBoost and LightGBM, significantly outperformed these systems, achieving AUROCs of up to 0.977. ML models were trained on 70 variables, including demographic and clinical data, and leveraged cohort-specific features to enhance predictive accuracy. Despite their superior performance, ML models either lack external validation or exhibited reduced generalizability when validated on external datasets, highlighting challenges in standardizing predictive tools across different clinical settings. By identifying key mortality predictors such as ventilator use, glucose levels, and infection indicators, ML approaches demonstrated their capacity for nuanced analysis. In addition, ML's ability to dynamically update with new data enhances its potential to adapt to evolving ICU practices. This study underscored the transformative potential of ML-driven digitization in improving patient prognosis in ICUs while emphasizing the need for further optimization to balance accuracy, adaptability, and generalizability (15).

A Turkish research team examined the impact of digitalizing nursing documentation on time management, costs, and patient care in ICUs, with implications for mortality rates. By transitioning paper-based forms to a digital format, nurses saved an average of 56 min per patient per day, significantly reducing documentation time and allowing more time for direct patient care. This shift was associated with a reduction in errors, improved data accessibility, and enhanced workflow efficiency. National projections indicated that the time saved across Turkiye's occupied ICU beds could result in significant financial savings and improved nurse workload distribution, indirectly benefiting patient care outcomes. The cited prior research demonstrated a 12% reduction in ICU mortality rates linked to EHRs and computerized physician order entry (CPOE) systems. The reduction was attributed to fewer medical errors, enhanced clinical decision-making, and streamlined processes. These findings underscored the transformative potential of digitalization in healthcare, particularly in high-stakes environments like ICUs, where improved efficiency and error reduction directly influence patient survival. The study advocates for broader adoption of digital tools in ICUs to further optimize outcomes while addressing challenges such as initial costs, data privacy, and standardization (16, 17).

Another study using the MIMIC-IV dataset (73,141 ICU stays from 50,934 patients) applied machine learning models, specifically random forest and XGBoost, to predict LoS based on data from the first 24 h of admission. These models effectively classified short stays, with random forest achieving the highest accuracy, though predictions for extended stays were hindered by data complexity and granularity limitations. A hybrid stepwise approach combining classification and regression improved short-stay forecasts but underscored ongoing challenges in modeling prolonged ICU admissions. The study illustrates how digitalization of ICU data supports dynamic, system-level decision-making to enhance resource management and streamline patient flow. However, the study emphasized the need for more sophisticated models, such as deep learning, and the integration of temporal data to enhance predictive accuracy (18).

### 3.2 Impact on clinical deterioration and complications

Digitalization in ICU care extends beyond mortality prediction to the proactive management of clinical deterioration and readmission risk. Through the integration of hospital information systems and predictive analytics, researchers have begun to identify key factors influencing ICU LoS, early readmission, and patient flow dynamics.

An Iranian study leveraging data mining on inpatient records (2019–2020) illustrates how digital infrastructures can elucidate the dynamics of patient flow and resource use across hospital subsystems, including ICUs. Findings revealed that ICUs exhibited markedly longer LoS than general wards, particularly among older and more critically ill patients. A decision tree model pinpointed patient age, ward type, and gender as key variables influencing LoS, with ICU stays most often in the range of 5–9 days. The deployment of automated hospital information systems was critical in enabling this granular analysis, highlighting how digitalization enhances the system's capacity to identify high-risk cohorts, optimize throughput, and target interventions that balance efficiency with quality care in intensive care environments (19).

Another research group focused on predicting early ICU readmissions (within the same hospital stay) using optimized and explainable ML models. Employing the MIMIC-III database, which included 28,557 adult ICU admissions, and excluded patients who died during the initial ICU stay, the researchers utilized the XGBoost algorithm combined with Bayesian optimization and Shapley Additive Explanations to improve model performance and interpretability. The optimized model achieved an AUROC of 0.92, outperforming prior state-of-the-art models. Key features influencing predictions included ICU length of stay, PaO₂ levels, and white blood cell counts. By identifying critical thresholds for these parameters, the model enabled the configuration of alarms for high-risk patients, facilitating timely interventions. Early readmission, linked to increased mortality, morbidity, and prolonged hospital stays, was predicted with high accuracy, addressing a critical need in ICU management. Digitalization and data-driven approaches significantly enhanced early detection, reducing preventable readmissions and improving resource allocation. The study underscores the transformative potential of explainable AI in critical care by supporting clinicians with actionable insights while addressing ethical concerns of black-box models. Future applications aim to validate these models in broader datasets and integrate them into real-time ICU monitoring systems for enhanced patient outcomes (20).

A previous literature review analyzed ICU readmission risk factors and patient outcomes, focusing on the use of the National Early Warning Score (NEWS) to reduce readmissions. Factors significantly influencing ICU readmission rates included respiratory distress or failure, accounting for 18%–59% of cases, followed by cardiac events (15%–30%). Patients with reduced Glasgow Coma Scale (GCS) scores, male sex, age over 54 years, and multiple comorbidities were at increased risk, with odds ratios as high as 22.3 for GCS < 9 and 8.4 for three or more comorbidities. Premature transfers due to ICU bed shortages often resulted in higher readmission rates, which were in the range of 1.5%–13.4% across studies. Readmitted patients experienced significantly increased hospital mortality (10–27.5%) and length of stay, ranging from an additional 11–40 days. The NEWS tool, which evaluates six vital signs, demonstrated high sensitivity (93.6%) and specificity (82.2%) for predicting deterioration within 24 h of ICU discharge, offering a promising strategy for mitigating avoidable readmissions. Digital tools like NEWS provide actionable insights for clinicians, supporting safer ICU discharge decisions and improving overall patient outcomes (5, 21).

### 3.3 Personalized care interventions

As ICU care extends beyond acute stabilization, digitalization increasingly plays a role in personalized monitoring and postdischarge recovery. Innovations such as wearable devices and digital recovery platforms aim to support patients through the continuum of care. These technologies hold promise for improving long-term outcomes, but also raise questions about data quality, usability, and equitable access.

A more recent study explored the feasibility and accuracy of using wearable devices for automatic calculation of the NEWS2 to monitor hospitalized patients in a tertiary care setting. Conducted in Switzerland, the study included patients who wore wristbands for continuous heart and respiratory rate monitoring over three days. NEWS2 introduces additional modifications to the original score (NEWS), including a dedicated scale for oxygen saturation tailored to patients with confirmed or suspected hypercapnic respiratory failure, such as those with chronic obstructive pulmonary disease (COPD). The automated NEWS2 calculations were compared to conventional measurements by medical staff. The results demonstrated substantial agreement between the two methods (Cohen's Kappa = 0.76), highlighting the potential of wearable devices for continuous monitoring. However, data quality challenges arose, particularly with respiratory rate measurements, which impacted accuracy. Patient acceptance of the wearable devices was high, with most participants appreciating the safety and convenience of continuous monitoring. Concerns primarily revolved around data privacy and device comfort. The study concluded that while wearables can enhance patient monitoring and reduce workload, further improvements in device accuracy and integration with clinical systems are needed to fully replace conventional methods (22, 23).

An observational study implemented at a large tertiary academic center in London, UK investigated the effectiveness of a digitally enabled ICU recovery pathway focused on personalized recovery goals for ICU survivors after discharge. A digital platform (aTouchAway) was used to facilitate patient engagement, the setting of recovery goals, and self-monitoring, aiming to address rehabilitation needs immediately after hospital discharge. Key results indicated that ICU survivors face substantial challenges in basic activities such as walking and self-care upon home discharge. Despite the individualized support, only approximately 50% of the recovery goals were achieved within the set timeframes, and the functional improvements were limited, highlighting ongoing rehabilitative needs for this patient category. Overall, the digital recovery pathway received positive feedback for acceptability, feasibility, and appropriateness, with moderate healthcare costs associated with outpatient and community services but minimal acute care needs. The study indicates that although digital platforms can support ICU recovery, challenges remain in addressing the full spectrum of survivors' needs, particularly in the physical and psychological domains. Future research could further explore this potential to lower healthcare costs through broader application and more extensive use of digital support tools for long-term ICU recovery (24).

## 4 The influence of ICU digitalization on medical care metrics

Infections remain a leading cause of ICU morbidity and mortality, making their early detection and management a critical priority. The integration of evidence-based practices by digital innovations is increasingly being leveraged to enhance diagnostic accuracy, optimize antimicrobial use, and support timely interventions. From sepsis prediction to infection-specific models, these tools are reshaping how critical care teams detect and respond to infectious threats in real time.

### 4.1 Integration of evidence-based practices

A comprehensive review evaluated the integration of AI and ML into infection management in the ICU, emphasizing its potential to enhance diagnostic accuracy, antimicrobial stewardship, and clinical decision-making. AI models have been developed to predict infections such as ventilator-associated pneumonia (VAP), central-line-associated bloodstream infections (CLABSI), and sepsis, enabling early intervention and reducing morbidity and mortality rates. Among the quoted researchers, Shimabukuro et al. demonstrated that an ML-based sepsis prediction model reduced mortality and ICU length of stay by predicting sepsis up to 4 h before clinical onset, with the ML algorithm demonstrating higher sensitivity and specificity (AUROC 0.952) compared to traditional scoring systems like SOFA, SIRS, and MEWS. Similarly, models like Moni-ICU have used fuzzy logic to identify infections in preclinical stages, offering significant time advantages over traditional rule-based systems. Diagnostic advancements have included combining chest X-rays with ML algorithms, as shown by Hwang et al., improving diagnostic performance for thoracic diseases, with a deep-learning algorithm (DLA) where, using a dataset of 89,834 radiographs, the DLA demonstrated high diagnostic accuracy with an AUROC of 0.979 in external validation, outperforming non-radiology physicians, general radiologists, and thoracic radiologists in both image-wise classification and lesion-wise localization. Another quoted study by Chen et al. reported on utilizing electronic nose sensors, combined with ML to diagnose VAP in ICU patients. By analyzing volatile organic compounds in exhaled breath from 33 VAP cases and 26 controls, the researchers developed prediction models using eight ML algorithms. AI also aids antimicrobial stewardship by optimizing empirical therapy decisions, as in the study by Feretzakis et al., which used ML to predict antibiotic susceptibility; by analyzing 888 clinical samples from 345 ICU patients over 2 years, the study leveraged eight ML models, including random forest and Multilayer Perceptron, achieving a ROC area of 0.726. The models used readily available microbiological data, such as Gram stain results, without requiring patient clinical data, ensuring a low-cost and accessible approach. By accurately predicting antibiotic resistance, the ML models guided early, tailored antibiotic therapy, reducing the time to initiate effective treatment and potentially limiting the spread of multidrug-resistant pathogens. Moreover, ML-supported tools help differentiate inflammation from infection, exemplified by Lamping et al.'s model for distinguishing sepsis from non-infectious systemic inflammation. Utilizing data from 238 SIRS and 58 sepsis episodes in a German pediatric intensive care unit (PICU), the researchers identified eight key predictors, including length of PICU stay before onset, interleukin-6 (IL-6), platelet count, procalcitonin, and C-reactive protein (CRP). The model, based on random forest algorithms, achieved an area under the curve (AUC) of 0.78 in the validation dataset, outperforming individual biomarkers such as CRP, IL-6, and procalcitonin alone. The model demonstrated the ability to classify all sepsis cases correctly while reducing unnecessary antibiotic use in non-infectious SIRS cases by 30%. All these innovations promise to reduce unnecessary antimicrobial use and enhance patient outcomes. Despite encouraging internal validation, the review highlights the need for external validation, addressing dataset shifts, and ensuring clinicians are trained to deploy and monitor these systems effectively (25–31). Comparative analysis of the selected studies is presented in Table 1.

**Table 1:** Comparative analysis of selected studies.

Study	Method	Models/tools	Outcomes	Limitations
Lam et al. (6)	Retrospective cohort analysis of ICU admissions over 8 years; all first admissions; risk adjustment via APACHE IV	APACHE IV model (scoring system)	Mean ICU LoS ∼5 days, median ∼2.5 days; ICU mortality ∼10%; hospital mortality ∼20%; standardized mortality ratio (SMR) ∼0.8; LOS ratio ∼1.0	Single center; excludes patients <16 and LOS <4 h; APACHE IV's calibration may drift over time; may not capture all severity or new therapies
Choi et al. (15)	Retrospective, large sample from two university hospitals; EHR data; comparison with traditional scoring	Several ML algorithms (XGBoost, etc.), trained on many clinical variables; compared with SAPS III, APACHE III	ML models achieved very high AUROC (≈0.977 in one hospital, ≈0.955 in the other), significantly outperforming classical scores	Performance drops across different hospitals; risk of overfitting; model complexity; requires extensive and high-quality EHR data; may be less interpretable for clinicians
Golestani et al. (9)	Retrospective cohort comparison of ICU management models (open vs. semiclosed) using hospital database; ∼1,064 patients analyzed	Analysis comparing ICU models (staffing structure) rather than advanced ML; statistical comparisons of LOS, bed disposition etc.	Found that semi-closed ICUs had shorter ICU stays and better bed-disposition metrics; staffing model was associated with resource utilization differences	Does not use predictive models or external validation; observational design limits causal inference; potential confounding (severity) not fully controlled
Belciug et al. (10)	Simulation/modeling study using queuing theory + evolutionary/genetic algorithm; real data from geriatric medicine department; what-if analyses	Queuing models + compartmental model + genetic algorithm to optimize bed allocation and resource utilization	Demonstrated that combining these methods can suggest improvements in bed occupancy, reduce waiting, improve resource utilization under various scenarios. Also flexible policy evaluation (“what-if”)	Does not directly measure patient outcomes; simulation rather than real-world trial; model assumptions (arrival patterns, service time distributions) may not generalize; data from non-ICU departments (geriatric) so may differ for ICU.
Yilmaztürk et al. (16)	Observational time-motion study + cost-analysis; comparing paper vs. digital forms in ICU of university hospital in Turkey; measured with volunteer nurses + retrospective form data over many patient-days.	Not ML; digital vs. paper forms, metrics of time saved per nurse per patient per day; projected cost savings.	Digitalization saved ∼56 min per nurse per patient per day (∼3.95%), reduced paper/printer costs; projected large annual cost savings for full ICU bed occupancy.	Only two volunteer nurses for direct measurement; possible bias in selection of forms; savings projected rather than measured in full scale; limited (single center, Turkiye).
González-Nóvoa et al. (20)	Retrospective study using MIMIC-III database; optimized ML with explainability techniques	XGBoost with Bayesian optimization; SHAP (Shapley Additive Explanations) for interpretability	Achieved AUROC ∼0.92 ± 0.03 for predicting early ICU readmission; exceeds many prior models (usually ∼0.66-0.78)	Same-hospital/same dataset design; risk of overfitting; may not generalize to units with different patient populations or resource levels; interpretability adds clarity but deployment & clinical integration remain challenging
Reichl et al. (22)	Prospective pilot study; hospitalized patients in an acute care hospital; vital signs collected continuously via wearable devices + hospital information system; automated calculation of NEWS2; compared automated vs. conventional measurement	Wearable devices measuring heart rate, respiratory rate, oxygen saturation, temperature, etc.; automatic scoring of NEWS2; continuous monitoring algorithm	Showed feasibility of automatically generating NEWS2 from wearables; improved detection of patient deterioration; demonstrated significant agreement vs. conventional manual measurement; helped identify errors or delays inherent in manual charting	Pilot scale: small sample size; only in acute care hospital (not ICU specifically); some vital signs (respiratory rate) less reliable from wearables; data quality and missing measurements issues; user comfort & integration concerns; not yet tested for impact on hard outcomes like mortality
Shimabukuro et al. (26)	Randomized controlled trial in two medical-surgical ICUs; adult patients; comparing usual sepsis detector vs. machine learning algorithm (MLA) + clinical team alerts; primary outcome length of stay, secondary outcome in-hospital mortality	MLA based on six vital signs; alerts triggered to care team; standard severe sepsis detection tools in control arm	Average length of stay reduced from ∼13.0 to ∼10.3 days (*p* = 0.042); in-hospital mortality decreased by ∼12.4 percentage points (*p* = 0.018) in experimental vs. control group; relative reduction ∼58%	Sample size moderate; open-label design (clinicians could know alerts); external validation limited; algorithm relies on alerting, which depends on how the care team responds; risks of false positives/alert fatigue not deeply assessed
De Bruin et al. (27)	Retrospective observational study in ICU/hospital setting; patient data streams assessed by fuzzy logic rules; includes ICU patient-days	Fuzzy logic based monitoring/decision support system; algorithms using non-crisp thresholds	Able to classify borderline infection instances, offering earlier detection compared to strictly dichotomous methods; helps in surveillance of healthcare associated infections (HAI); presented frequencies of “borderline” classification; demonstrated that fuzzy logic detects mild or partial signs earlier	The definition of “borderline infection” is vague; performance vs. gold standard infection definitions variable; potential false positives; retrospective design; how well it works in real-time clinical workflow or alerts to clinicians not fully tested; resource intensity of continuous data collection
Hwang et al. (28)	Diagnostic/retrospective study; large dataset of chest radiographs; trained on single center, external validation in multiple institutions; performance tested vs. radiologists.	Deep learning algorithm (ResNet-based convolutional neural network, etc.), lesion-wise localization and image-wise classification; handles multiple thoracic disease categories	Very high AUROC for classification across external validation datasets (∼0.97-0.98); outperformed physicians alone; assistance by algorithm improves sensitivity/specificity; improves speed/timeliness of reporting especially in urgent/critical cases	Rx-based, not necessarily ICU-patient specific; image quality, dataset diversity issues; risk of overfitting; external validation is good but may still generalize less to settings with different imaging equipment or patient mix; clinical impact (on outcomes, not just diagnosis) less clear; potential for false positives and burden on radiology workflow

### 4.2 Standardization of clinical workflow

Digitalization is increasingly used to streamline ICU workflows by embedding AI-driven tools into diagnostic and therapeutic decision-making processes. From early risk prediction in sepsis-associated acute kidney injury (SA-AKI) to standardized diagnostic support for conditions like pneumothorax and neonatal sepsis, these technologies aim to offer scalable, real-time solutions.

Another recent review highlighted the transformative role of AI and ML in managing SA-AKI, a severe complication in critically ill patients associated with high mortality rates. Traditional methods, including statistical models and biomarkers, have been foundational but face limitations like delayed diagnostics and inconsistent sensitivity. AI and ML overcome these barriers by identifying complex patterns within large datasets, offering superior predictive accuracy and real-time risk assessment. Techniques, such as supervised learning with models like XGBoost and RNN-LSTM (Recurrent Neural Network-Long Short-Term Memory), have shown exceptional performance, achieving high AUC values (up to 1.0 in a study using RNN-LSTM) for early detection and mortality prediction. Among the quoted works, research by Rank et al. demonstrated AI's ability to outperform clinicians in predicting AKI onset using electronic health records focuses on a deep-learning-based approach to predict AKI after cardiothoracic surgery, with significant implications for managing associated complications, including sepsis. A RNN was developed by Rank et al., for real-time EHR data analysis to predict AKI up to 7 days postoperatively. This model achieved an area under the curve (AUC) of 0.89–0.90, outperforming experienced clinicians (AUC = 0.75) by providing earlier and more accurate predictions. The RNN utilized a wide range of clinical parameters, including laboratory results, hemodynamic data, and nephrotoxic drug administration, enabling dynamic, personalized risk assessments. The authors emphasize that early detection of AKI is critical in preventing downstream complications such as sepsis, as AKI contributes to systemic inflammation and organ dysfunction. Integrating such predictive tools into hospital workflows could facilitate earlier nephrologist involvement and implementation of preventive measures (e.g. optimizing hemodynamics and avoiding nephrotoxic agents), thereby improving patient outcomes. However, the paper also acknowledges limitations such as the need for broader validation across patient cohorts and challenges in adapting retrospective models for real-world clinical settings. The RNN's high sensitivity and calibration hold promise for real-time, automated sepsis prevention strategies, complementing traditional physician-led care. Another cited research, by Luo et al., demonstrated how ML could differentiate transient from persistent AKI in sepsis patients, aiming to enhance early intervention strategies. Persistent AKI, often seen in SA-AKI, is associated with worse outcomes, including higher mortality rates and long-term complications. The study leveraged data from the MIMIC-III database, applying models such as artificial neural networks (ANN), logistic regression, and extreme gradient boosting to predict AKI persistence using routine clinical variables. Among these, the ANN and logistic regression models achieved the highest predictive accuracy, with an AUC of 0.76. The authors developed a simplified 14-variable risk prediction model to facilitate real-world application, integrating factors such as serum creatinine levels, urine output, mechanical ventilation, and renal replacement therapy (RRT) initiation. Early identification of patients with a high risk of persistent AKI can guide fluid management, optimize RRT timing, and improve sepsis-related outcomes by mitigating renal and systemic complications. However, the study acknowledges limitations, including its single-center design and the need for external validation to ensure the model's generalizability. This work underscores the potential of ML in refining sepsis management through personalized risk stratification and targeted therapeutic interventions. Unsupervised learning methods, such as unsupervised clustering employed by Lai et al. have revealed distinct SA-AKI sub-phenotypes, enabling tailored interventions in patients with SA-AKI requiring dialysis. The most critical findings emphasized the role of pre-dialysis hyperlactatemia as an independent predictor of poor outcomes, including increased mortality and decreased likelihood of renal recovery. Cluster 1 patients, characterized by severe acute illness despite younger age and lower comorbidity scores, exhibited the highest mortality rates (73.86%) and the lowest probability of becoming independent of dialysis. This sub-phenotype highlighted the disproportionate impact of acute illness severity on outcomes compared to baseline health status. The study proposed a clinical model incorporating 11 variables for early identification of high-risk patients, which demonstrated robust predictive performance (C-statistic = 0.99). These findings offer a framework for personalized sepsis management, suggesting that early recognition of high-risk sub-phenotypes and addressing factors such as hyperlactatemia could refine therapeutic interventions and improve patient outcomes. Future research is encouraged to validate these findings and explore biomarker-guided strategies for precision care in SA-AKI. The review advocates for integrating AI into SA-AKI care pathways to enhance precision medicine and streamline clinical workflows, offering a paradigm shift in early detection and personalized treatment. Despite these advances, challenges remain, including data privacy, algorithmic bias, and interpretability. Future directions emphasize federated learning, explainable AI, and real-time monitoring to maximize AI's potential in critical care settings (32–37).

AI models were developed in another study aiming to predict late-onset sepsis (LOS) in preterm infants using vital signs data from neonatal intensive care unit (NICU) monitors. Data from 492 infants born before 32 weeks of gestation were analyzed, extracting 102 features from heart rate (HR), respiratory rate, and oxygen saturation (SpO_2_) sampled at 1 Hz. The best-performing model, based on gradient boosting, achieved a high predictive accuracy with the best AUC of 0.875, detecting risk of LOS 6 h before clinical suspicion. To optimize clinical utility, the study tested alarm policies, including multithreshold alarms, which reduced false positives while maintaining high sensitivity (up to 96.1%). The model demonstrated that changes in features like HR-SampAsy (asymmetry), HR-SampEn (entropy), and HR-SpO_2_ cross-correlation were strongly associated with LOS, offering insights into the progression of sepsis. Compared to earlier methods using raw waveform signals, this approach yielded comparable accuracy with lower computational demands, enhancing applicability in diverse clinical settings. However, challenges like alarm fatigue and limited dataset size were noted. Future directions include validation in larger populations and developing new features to refine predictions. This study highlighted the potential of AI for early sepsis detection and risk factors identification, through vital signs monitoring, thus improving NICU care (38).

Another investigation conducted on 75 patients with dyspnea in an ICU compared the diagnostic efficacy of AI-assisted lung ultrasound to bedside X-ray for detecting pneumothorax in critically ill patients, using chest computed tomography (CT) as the gold standard. The study utilized the BLUE-plus protocol for lung ultrasound, which was enhanced with AI to recognize pleural lines and identify pneumothorax-related signs such as the absence of pleural slip or the presence of lung points. The results showed that AI-assisted ultrasound achieved a sensitivity of 79.4% and specificity of 85.4%, while bedside X-ray had a sensitivity of 82.4% and specificity of 80.5%. Although both methods displayed comparable diagnostic accuracy, lung ultrasound was significantly faster, with an average examination time of 5.3 min compared to 12.6 min for X-ray. Limitations of the ultrasound method included occasional false positives in conditions like pleural adhesions and acute respiratory distress syndrome (ARDS) and the influence of operator skill. The study highlighted the potential of AI to improve diagnostic efficiency while acknowledging the need for further validation in larger cohorts and optimization of AI models to enhance accuracy. The authors concluded that AI-assisted lung ultrasound is a promising alternative to X-ray for pneumothorax diagnosis in the ICU, offering speed and comparable diagnostic performance with the advantage of real-time, non-invasive imaging (39).

## 5 ICU digitalization effects on resource utilization

Effective resource management is essential in critical care, where demands often exceed capacity. Digital transformation (DT) and SmartICU frameworks have introduced real-time data integration, predictive analytics, and automation. They aim to streamline workflows and optimize the use of beds, equipment, and staff. These technologies could help reduce delays, enhance coordination, and ensure that critical resources are available when and where they are needed most.

### 5.1 Real-time resource management

Improved resource utilization and enhanced patient safety are cornerstones of the SmartICU concept. SmartICU systems use real-time data and predictive analytics to allocate resources more effectively. This includes optimizing the use of ICU beds, ventilators, and medical supplies, reducing waste, and ensuring that resources are available when needed. SmartICUs utilize technologies such as predictive analytics to anticipate complications, automated alarms to alert healthcare professionals of changes in patient conditions, and integrated platforms to consolidate and analyze patient data from various sources. This approach aims to improve patient outcomes, optimize resource allocation, and reduce human error (40).

A case study from a large philanthropic hospital in São Paulo detailed the implementation of digital transformation (DT) in bed management. The hospital, equipped with 529 operational beds, including critical care and semi-intensive units, undertook DT to address inefficiencies in bed allocation and utilization. Before digitalization, the process relied heavily on manual workflows, leading to delays, underutilized resources, and miscommunication between departments. After digitalization, the hospital integrated systems like Enterprise Resource Planning, specialized hygiene and maintenance software, and real-time dashboards for bed tracking. These technologies streamlined the workflow by automating bed turnover, aligning discharge timings with cleaning schedules, and enhancing communication between housekeeping and maintenance teams. DT significantly improved bed turnover rates, reducing the average replacement interval from 1.7 days to near real-time synchronization, allowing faster patient admissions. The transformation yielded measurable organizational benefits, including higher process efficiency, better resource allocation, and increased staff satisfaction. Patient satisfaction improved due to reduced waiting times, particularly in emergency and surgical care. However, challenges persisted, such as resistance to change, high initial costs, and gaps in system integration. Notably, the study emphasized the importance of aligning technology with organizational culture and staff training to maximize DT benefits. This case demonstrated the potential of DT to optimize ICU and general bed management, showcasing its capacity to alleviate systemic bottlenecks and improve healthcare delivery in complex hospital environments (41).

### 5.2 Staffing and workload redistribution

Real-time resource management can extend to human resources, with staff time and clinical attention allocation in critical care. As digital tools help streamline bed turnover and admission processes, they also impact workforce dynamics, by shifting workloads, modifying care delivery locations, and supporting early interventions.

A review explored interventions aimed at improving patient flow within adult ICUs, with a focus on reducing admission delays, discharge delays, and after-hours discharges. The researchers found that various interventions, including workflow modifications, decision support tools, and enhanced communication practices, positively impacted ICU processes. Organizational-level strategies, such as protocols and alert systems, were particularly effective in facilitating timely discharges. However, few interventions directly addressed delayed admissions, and only a minority of studies showed significant improvements in patient outcomes, such as reduced mortality or length of stay, following these interventions. Thus, Aletreby et al. (43), Hsieh et al. (44), and O’Callaghan et al. (45) evaluated delayed admissions impact. The first study, on a single-center retrospective cohort at King Saud Medical City, Riyadh, revealed that delayed admission was associated with a 2.6 times higher ICU mortality risk [95% confidence interval (CI) 1.9–3.5; *p* < 0.001], while delays beyond 2 h began to affect outcomes, with the strongest association seen after 4 h. Contributing factors included ED overcrowding, resource constraints, and complex patient conditions requiring time-intensive diagnostic and therapeutic interventions. The second study found that patients experiencing delayed ICU admission (>1 h) exhibited significantly higher in-hospital mortality rates (26.6% overall, with higher rates correlated to prolonged delays). Kaplan–Meier survival curves demonstrated a clear survival disadvantage for delayed admission patients compared to those admitted within the optimal 1-h window. ICU delays also correlated positively with prolonged hospital stays, longer ICU stays, and extended mechanical ventilation periods, highlighting their compounding impact on patient outcomes and resource utilization. The third study, conducted at Charing Cross Hospital, found that delayed ICU admission did not significantly affect ICU or hospital mortality rates or length of ICU stay. However, delayed patients exhibited a significantly higher requirement for advanced respiratory support (92.3% vs. 76.4%) and longer durations of mechanical ventilation (median of 4 vs. 3 days). The concept of “critical care without walls,” where ICU-level care is provided in non-ICU settings while awaiting admission, emerged as a viable strategy to address bed shortages. The review findings also underscore the role of telemedicine in improving mortality and length of stay outcomes. To better address patient flow challenges, future studies should focus on implementing multidisciplinary communication and digital technologies while assessing intervention effectiveness through controlled trials (42–45).

## 6 Digitalization impact on ICU patients and family satisfaction

Beyond clinical metrics, ICU digitalization plays a growing role in enhancing patient and family experience through personalization and engagement. From goal-oriented recovery platforms to digitally enriched ICU environments, emerging tools empower patients, support family involvement, and promote emotional well-being. These innovations not only improve satisfaction but may also contribute to better functional and psychological outcomes during and after ICU stays.

### 6.1 Patient engagement and personalized feedback

An observational study explored the implementation of a novel digital recovery pathway for ICU survivors, emphasizing personalized recovery goal-setting to address functional, psychological, and social challenges after critical illness. The patients appreciated the pathway's accessibility, reporting high acceptability and feasibility scores. Although healthcare costs were moderate, with a median cost of £784 per patient, participants accessed outpatient and community services more frequently than acute care. The program also focused on improving patient and family satisfaction by involving patients in shared decision-making and tailoring interventions to individual needs (24).

A patient-centered ICU transformation was demonstrated in another study, specifically focusing on how these changes enhance patient and family satisfaction. By addressing common ICU stressors such as noise, poor lighting, and limited family engagement, the redesign incorporated advanced digital technologies to create a more personalized and calming environment. Key features included circadian lighting systems mimicking natural daylight, virtual windows displaying dynamic nature views, and integrated entertainment systems allowing patients to control audio-visual content. These interventions not only reduced sensory overload and anxiety for patients but also improved their emotional well-being by fostering autonomy and comfort. For families, virtual visiting platforms played a critical role, particularly in scenarios of restricted physical access, enabling meaningful communication with patients. Feedback from patients and families showed significant improvements in satisfaction, citing reduced stress, enhanced emotional connection, and a more healing environment. In addition, these upgrades contributed to measurable clinical benefits, such as reduced delirium rates and improved sleep quality, which are vital for recovery. This study underscores the potential scalability of these digital interventions, advocating for broader implementation to improve both patient and family experiences in diverse ICU settings, while recommending further studies to assess long-term impacts on clinical and psychological outcomes (46).

### 6.2 Psychosocial support

Building on efforts to personalize care and foster engagement, digital tools also play a vital role in addressing the emotional and psychosocial needs of ICU patients and their families. Especially during crises like the COVID-19 pandemic, virtual communication platforms have emerged as essential tools to reduce isolation, sustain emotional connection, and humanize care when physical access is limited.

An innovative digital initiative called “Remote Family Conference and Patient Visits in the COVID Hospital” was implemented at the AOU Policlinico of Bari during the COVID-19 pandemic. The project aimed to mitigate the emotional and informational challenges faced by families of critically ill patients due to restricted visitation policies. The initiative used Information and Communications Technology to facilitate communication between patients, families, and healthcare teams, thus improving the humanization of care under pandemic constraints. A satisfaction survey administered to 19 families highlighted excellent ratings for communication quality and staff courtesy, with families reporting significant alleviation of emotional distress due to continuous updates and contact. The digital approach ensured consistent communication, reduced isolation, and provided a structured platform for family engagement, emphasizing its potential to transform healthcare delivery during crises (47).

## 7 Digitalization influence on ICU quality and safety indicators

Although patient-centered innovations improve satisfaction and engagement, the broader impact of ICU digitalization is equally reflected in quality and safety metrics. Care quality and safety represent the cornerstone of all ICU processes, as it is a sine qua non-condition for all the other indicators. Technologies that support infection control, medication accuracy, and early risk identification not only improve individual outcomes but also strengthen system-wide reliability.

### 7.1 Infection control and adverse events

Recent data from Brazilian researchers showcase the integration of digital technologies into infection prevention and control strategies within ICUs, with the development of a series of educational videos designed to involve patients and their families in infection prevention processes, promoting a collaborative care model. The research underlined the importance of family involvement in healthcare, supported by evidence that patient outcomes improve when families are engaged. These videos focused on educating viewers about infection control measures such as hand hygiene, prevention of ventilator-associated pneumonia, and catheter-related infections. Developed through a rigorous methodology involving ICU nurses, the videos were tailored to provide clear, accessible information, enhancing understanding of ICU care routines and infection risks. By leveraging multimedia elements, the videos aimed to bridge communication gaps, especially for those with limited health literacy. The study also highlighted the potential of such digital tools to complement existing infection control protocols, fostering a culture of safety and reducing healthcare-related infections. Bechman et al. evaluated a semiautomated contact tracing tool (CTT), based on Microsoft Excel and Visual Basic, implemented in a German tertiary hospital to enhance the management of COVID-19 exposure among healthcare workers. The study highlighted the role of the CTT in identifying staff contacts related to 322 COVID-19–positive index cases in 2021, offering a structured workflow that included automated PCR testing appointments and real-time dissemination of hygiene instructions based on risk categories. The findings underscored the efficiency of digital tools in infection control, demonstrating a 75% reduction in time requirements for contact tracing compared to previous paper-based methods. Despite minor technical challenges, the CTT was strongly preferred by infection control staff for its ability to automate key processes, reduce administrative burdens, and improve data reliability. Domegan et al. examined an outbreak of OXA-48 carbapenemase-producing *Enterobacterales* in an Irish hospital between 2018 and 2019, using a combination of social network analysis (SNA) and genomic tools to trace the transmission dynamics. The outbreak involved 45 patients, primarily elderly with complex comorbidities, and was linked to extended hospital stays, high bed occupancy, and suboptimal ward infrastructure. Despite infection prevention and control measures like screening, hydrogen peroxide vapor decontamination, and isolation, the outbreak persisted, with three distinct temporal clusters observed. Genomic analysis identified seven species of *Enterobacterales*, with a particular clonal group (ST78) becoming predominant. SNA revealed key transmission links and “super-spreader” patients with behavioral issues or frequent intraward movements. The findings emphasize the importance of integrating SNA with genomic data early in outbreaks to map connections, identify hotspots, and implement targeted interventions (48–50).

Autonomous units assisted by digital tools can also have an impact on ICU infections. Worlikar et al. explored the feasibility of employing a humanoid robot, DAVE, to improve hand hygiene adherence in a hospital setting, addressing the significant issue of healthcare-associated infections. Conducted in a tertiary hospital in Ireland, the study utilized DAVE as a single-intervention reminder at the main hospital entrance and diabetes outpatient department. DAVE engaged individuals through audio and visual prompts, including educational videos, encouraging the use of alcohol-based hand rubs. The intervention yielded a 29% overall increase in hand hygiene compliance, significantly higher than traditional reminder methods like posters and light cues, which typically achieve modest improvements in the range of 7%–8.5%. The study also highlights the importance of implementing novel technologies like humanoid robots in fostering adherence to essential practices, particularly in healthcare environments where direct transmission via colonized hands is common. Potential challenges, such as DAVE becoming an infection source or being disruptive, necessitate further research to refine its use. If successful in clinical applications, DAVE could serve as a dual-purpose tool for both promoting hand hygiene and auditing compliance using AI. This study represents a novel step toward integrating robotics in infection prevention strategies. Another study examined the efficacy of an Autonomous Sanitary Sterilization Ultraviolet Machine (ASSUM), a UV-C emitting robot, for terminal disinfection in surgical theaters and ICUs, over a period of 10 months, at a tertiary hospital. The ASSUM was specifically targeting environments where patients with multidrug-resistant (MDR) microorganisms were treated. The results showed that although the ASSUM robot effectively reduced bacterial counts on ICU and theater surfaces, this reduction did not significantly impact SSI rates or MDR acquisition rates in clinical samples. The bacterial burden, however, was markedly lower after intervention, particularly in ICU settings, demonstrating ASSUM's capacity for effective surface sterilization beyond standard manual cleaning. Despite the reduction in environmental bacteria, the study concluded that using the UV-C robot did not lead to a measurable decrease in infection rates, suggesting that other factors, such as personnel hand hygiene and other environmental reservoirs, may play a larger role in infection transmission in clinical settings. Thus, the cost benefits of ASSUM would be maximized when used alongside comprehensive infection control practices rather than as a standalone solution (51, 52).

### 7.2 Medication safety and compliance

Another recent research, the TIME study, evaluated the transition from a paper-based system to a fully digital hospital in two wards, incorporating systems like computerized physician order entry, clinical decision support systems, electronic health records, and automated medication dispensing systems, which significantly reduced medication errors. The average number of voluntarily reported medication incidents and the rate of prescribing errors decreased. Procedural and dosing errors were also diminished, indicating the impact of digitalization on improving adherence to safer prescribing practices. These systems helped minimize errors through safety features such as allergy and drug interaction alerts, dose calculators, and integrated dashboards for real-time analytics. However, therapeutic errors did not see a significant change, reflecting variability in how dedicated systems influence clinical decision-making. Despite the reductions in errors, the study noted that digitalization introduced challenges such as alert fatigue, workflow inefficiencies, and clinician burnout, emphasizing the need for ongoing training and optimization of digital tools (53).

A French study evaluated compliance with guidelines for preventing central venous catheter (CVC)-related infections in two university hospitals, where a multicenter observational audit examined adherence to infection prevention protocols using a digital tool. The key findings included a 90% compliance rate with hand hygiene prerequisites, though adherence to repeated hand hygiene before infusion was lower at 59%. Compliance with the proper rinsing technique was 75.6%, while sterile glove usage and other critical steps varied. The study identified substantial gaps, such as low rates of identity verification and patient explanation of procedures. Observations revealed differences in protocol adherence between ICU and non-ICU wards, influenced by factors such as patient cognitive status. Although compliance with guidelines was satisfactory overall, significant discrepancies persisted, highlighting barriers like insufficient awareness and inconsistent practices. The study underscored the importance of audits in identifying gaps and enhancing infection control measures to reduce complications associated with CVCs (54).

VAP is considered a significant ICU safety-related incident and the most frequent nosocomial infection, associated with extended ICU stays and higher healthcare costs and mortality rates. VAP remains a critical challenge in ICUs, due to its high prevalence, complexity, and diagnostic ambiguities. Giang et al. evaluated ML methods such as gradient-boosted trees and logistic regression, demonstrating their superior performance over traditional clinical scoring tools like CURB-65 and PIRO in predicting VAP both after ICU admission and after intubation. These models utilize accessible patient data, such as duration of mechanical ventilation, antibiotic use, sputum tests, and GCS scores, achieving high predictive performance, with AUROC values exceeding 0.85 in some cases. Importantly, ML methods can provide early alerts for high-risk patients, enabling timely clinical interventions and potentially preventing VAP by informing decisions about non-invasive ventilation strategies. The integration of ML also supports better antibiotic stewardship by reducing diagnostic delays and the risk of overtreatment. However, challenges such as model interpretability, reliance on retrospective data, and limited generalizability across institutions highlight the need for further validation and refinement (55–57).

A recent investigation evaluated the use of mobile dynamic digital radiography (M-DDR) for bedside assessment of silent aspiration (SA) in elderly ICU patients. The researchers evaluated the effectiveness of default grayscale images (DGI) and inverted grayscale images (IGI) obtained through M-DDR in identifying SA, using the Penetration-Aspiration Scale. Kappa coefficients indicated almost perfect agreement between DGI and IGI evaluations. The study found no significant diagnostic advantage between IGI and DGI, suggesting that DGI alone can be reliable for rapid bedside assessment. Although the approach showed promise, limitations included the reduced frame rate, which may slightly affect diagnostic precision. The study concluded that M-DDR enables feasible, efficient, and accurate bedside evaluation of swallowing function in critically ill elderly patients, potentially aiding in the standardization of swallowing management protocols in ICU settings. Future research should address optimizing imaging conditions and integrating real-time assessment capabilities (58).

### 7.3 Predictive analytics

Ventilation performance is another essential component for ICU patients' safety and prevention of complications such as VAP. The integration of intelligent monitoring systems into ventilation support management in ICUs demonstrates substantial improvements in clinical outcomes by enabling early detection and intervention for ventilator-associated events (VAEs). These systems, embedded within electronic medical records, continuously analyze patient data and automate alerts based on predefined VAE criteria, such as changes in positive end-expiratory pressure (PEEP) and inspired oxygen fraction (FiO2). The study by Liu et al. found that patients monitored with intelligent systems experienced earlier VAE detection, shorter ventilator days, reduced antibiotic use, and lower 14-day mortality rates. Moreover, the automated systems increased the detection of possible VAP by 17.1%, emphasizing their capability to identify risks that manual monitoring may miss. These improvements stem from the systems' ability to reduce human error, eliminate observer variability, and provide real-time data, which facilitates timely clinical interventions. Stivi et al. explored the transformative role of AI and ML in optimizing mechanical ventilation weaning processes, especially in patients with ARDS. Digitalization in ventilation support has enabled real-time data integration, improving prediction accuracy for successful weaning. Advanced ML models analyze a range of physiological parameters, such as tidal volume, respiratory rate, and heart rate, to assess patient readiness for weaning. These models, including neural networks and decision-tree algorithms, exhibit high predictive accuracy, with some achieving an AUROC of up to 0.98. This technology significantly reduces extubation failures, ventilation duration, and ICU length of stay by providing personalized, evidence-based recommendations. AI systems, like adaptive support ventilation (ASV) and SmartCare, also automate weaning protocols, adjusting ventilator settings dynamically based on patient responses. Lastly, a review evaluated the INTELLiVENT-ASV, a closed-loop mechanical ventilation system designed to enhance ICU ventilation management through digital automation. The findings indicated that INTELLiVENT-ASV improves ventilation effectiveness by maintaining lung-protective tidal volumes, optimizing oxygenation, and dynamically adjusting ventilator settings based on real-time physiological data such as end-tidal carbon dioxide and oxygen saturation levels. Studies consistently demonstrated that this system performs at least as effectively as conventional methods, with significant advantages in maintaining optimal ventilation parameters and reducing time spent in unsafe ventilation ranges. The automated adjustments in tidal volume, respiratory rate, and PEEP reduced manual intervention and workload for ICU staff, a critical advantage during high-demand periods like the COVID-19 pandemic. In addition, INTELLiVENT-ASV facilitated smoother transitions between passive and active patient states, improving patient-ventilator synchrony and supporting timely extubation readiness assessments. However, the review noted a lack of clear superiority in primary clinical outcomes such as reduced LoS or mortality, underscoring the need for larger, more targeted studies (59–62).

## 8 Discussion and conclusions

Digital tools, particularly ML models integrated with EHRs, have the potential to significantly enhance the prediction of in-hospital mortality in ICU settings. These tools outperform traditional scoring systems like APACHE III and SAPS III, achieving AUROC values as high as 0.977. By dynamically analyzing real-time data such as ventilator usage, glucose levels, and infection indicators, ML models can provide nuanced insights that adapt to evolving ICU practices. This results in more accurate risk assessments, enabling timely clinical interventions that improve survival rates. Although these tools enhance prognostic precision and patient outcomes, challenges related to their generalizability across diverse clinical settings emphasize the need for standardized and adaptable solutions.

The digital transformation of nursing documentation through EHRs and computerized physician order entry systems has markedly improved workflow efficiency and patient care in ICUs. By reducing documentation time, these tools would allow nurses to allocate more time to direct patient care, contributing to a reduction in medical errors and improving adherence to safety protocols. This transition has been linked to a 12% reduction in ICU mortality rates, highlighting the direct impact of streamlined processes on patient survival. Furthermore, enhanced data accessibility improves decision-making and workflow efficiency, addressing the high-stake demands of ICU environments.

In addition, ML tools, such as random forest and XGBoost algorithms, are aimed at predicting ICU LoS based on early vital signs, demographic information, and lab results. These models optimize resource allocation by identifying patients at risk of prolonged ICU stays, facilitating better planning and patient flow. Although the models are accurate for short stays, challenges remain in predicting longer stays due to data granularity and complex influencing factors. Dynamic modeling enabled by digitalization aids real-time decision-making, improving resource efficiency and enabling tailored interventions to reduce unnecessary LoS without compromising care quality.

The integration of ML algorithms like XGBoost combined with Bayesian optimization could also support the early detection of ICU readmission risks. By leveraging real-time clinical data, these tools accurately identify high-risk patients (AUROC of 0.92), enabling clinicians to configure alarms and prioritize early interventions. This may reduce preventable readmissions, which are often linked to increased mortality and extended hospital stays. By addressing critical thresholds for variables such as PaO₂ levels and white blood cell counts, ML-driven approaches can enhance resource utilization and optimize patient outcomes, as an example of the transformative potential of data-driven ICU management.

Digital tools have advanced infection control and ventilation management in ICUs. AI-enabled monitoring systems detect VAEs and predict VAP with accuracy, providing early warnings for timely interventions. These systems can also improve antibiotic stewardship by reducing over-treatment and diagnostic delays. Moreover, closed-loop ventilation systems like INTELLiVENT-ASV dynamically adjust ventilator settings based on real-time physiological data, maintaining lung-protective tidal volumes and optimizing oxygenation. Although some challenges remain, INTELLiVENT-ASV exemplifies the potential of digitally enabled tools to streamline ICU ventilation support, optimize resource utilization, and enhance patient care through real-time, data-driven decision-making.

Despite promising advancements, the application of artificial intelligence in intensive care settings faces several limitations. Many current machine learning models are trained on single-center datasets, which raises concerns about generalizability and external validity. Overfitting to local patient populations or documentation practices can lead to poor performance in other clinical environments. Furthermore, the lack of transparency in model architecture, commonly referred to as the “black box” problem, can hinder clinical trust and integration into real-time decision-making. Legal, ethical, and regulatory uncertainties also remain, especially around liability in AI-driven interventions.

Future research should prioritize prospective, multicenter validations of AI tools, ideally through randomized controlled trials where feasible. Developing interpretable models that align with clinician workflows will be essential for adoption. Moreover, collaborative efforts across institutions and disciplines can support the creation of standardized datasets and ethical frameworks for deployment. Investigations should also explore the integration of AI with patient-facing technologies and post-ICU recovery pathways, ensuring that digital innovations extend beyond acute care to support long-term outcomes.
